# Gastroesophageal varices evaluation using spleen‐dedicated stiffness measurement by vibration‐controlled transient elastography

**DOI:** 10.1002/jgh3.12689

**Published:** 2021-12-14

**Authors:** Koki Nagai, Yuji Ogawa, Takashi Kobayashi, Michihiro Iwaki, Asako Nogami, Yasushi Honda, Takaomi Kessoku, Yusuke Saigusa, Kento Imajo, Masato Yoneda, Hiroyuki Kirikoshi, Tatsuji Komatsu, Satoru Saito, Atsushi Nakajima

**Affiliations:** ^1^ Department of Gastroenterology and Hepatology Yokohama City University Graduate School of Medicine Yokohama Japan; ^2^ Department of Gastroenterology Shin‐yurigaoka General Hospital Kawasaki Japan; ^3^ Department of Gastroenterology National Hospital Organization Yokohama Medical Center Yokohama Japan; ^4^ Department of Biostatistics Yokohama City University School of Medicine Yokohama Japan; ^5^ Department of Clinical Laboratory Yokohama City University Hospital Yokohama Japan

**Keywords:** gastroesophageal varices, hepatic venous pressure gradient, liver stiffness measurement, portal hypertension, spleen stiffness measurement

## Abstract

**Background and Aim:**

Liver stiffness measurement (LSM) and spleen stiffness measurement (SSM@50 Hz) using standard vibration‐controlled transient elastography (VCTE) have been studied as a noninvasive test for screening of gastroesophageal varices (GEV) in chronic liver disease (CLD). Recently, a novel spleen‐dedicated VCTE (SSM@100 Hz) has been developed. We evaluated the diagnostic performance of SSM@100 Hz, SSM@50 Hz, LSM, and other noninvasive tests using esophagogastroduodenoscopy (EGD) as the reference as well as the correlation with hepatic venous pressure gradient (HVPG).

**Methods:**

A total of 123 patients with CLD enrolled in this cross‐sectional study. SSM@100 Hz, SSM@50 Hz, and LSM were determined by VCTE. EGD and HVPG were performed within 12 weeks before or after VCTE.

**Results:**

GEV were present in 60 patients. Failure or suboptimal SSM were fewer at 100 Hz (4.0%) than at 50 Hz (17.7%). All SSM values obtained at 100 Hz were lower than the 100 kPa ceiling threshold, but 10 patients reached the 75 kPa ceiling threshold for SSM@50 Hz. SSM@100 Hz was most accurate (area under the receiver operating characteristic [AUROC] = 0.944) for the diagnosis of GEV compared to SSM@50 Hz, LSM, and scoring systems. AUROC of SSM@100 Hz for diagnosis of high‐bleeding risk varices (HRV) was 0.941, which was significantly higher than that of SSM@50 Hz (AUROC = 0.842, *P* = 0.002). SSM@100 Hz showed higher specificity (82.0%) for diagnosis of HRV than SSM@50 Hz (specificity = 67.1%). SSM@100 Hz was significantly correlated with HVPG (*r* = 0.71, *P* < 0.001).

**Conclusions:**

The novel spleen‐dedicated VCTE examination can be used for noninvasive assessment of GEV and HVPG in CLD. Japan Registry of Clinical Trials Registry No. jRCTs032200119.

## Graphical abstract

The novel spleen‐dedicated vibration‐controlled transient elastography examination can be used for noninvasive assessment of gastroesophageal varices and hepatic venous pressure gradient in chronic liver disease.

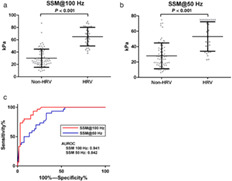

## Introduction

Gastroesophageal varices (GEV) are mainly induced by portal hypertension,^1^ which is one of the most common consequences of chronic liver disease (CLD). In patients with compensated cirrhosis, GEV are present in 30–40%, whereas they can be present in up to 85% of patients with decompensated cirrhosis.^1^, ^2^ Variceal hemorrhage occurs at a rate of around 10–15% per year,^3^, ^4^ and mortality is still as high as 15–20%.^5^ Esophagogastroduodenoscopy (EGD) is the best method for the diagnosis of GEV, and allows the identification of additional signs used to stratify bleeding risk (size of varices, presence of red color signs, and wale marks).^6^ However, the invasive nature of EGD leads to significant healthcare costs and patient discomfort.^7^

Portal hypertension contributes to the development of GEV.^8^ Measuring the hepatic venous pressure gradient (HVPG) via hepatic vein catheterization reliably evaluates the portal hypertension. Clinically significant portal hypertension (CSPH), defined by an HVPG ≥10 mmHg is associated with an increased risk of developing varices and overt clinical decompensation in the form of variceal hemorrhage, ascites, and hepatic encephalopathy.^1^, ^8^, ^9^

Since the diagnoses of GEV and CSPH require EGD and hepatic vein catheterization, which are invasive and require specific expertise, there is need for noninvasive methods. Stiffness measurement by vibration‐controlled transient elastography (VCTE) was introduced in 2003 specifically for the liver with fixed shear wave frequency at 50 Hz, specifically adjusted for a certain measurement depth and a stiffness range between 1.5 and 75 kPa. Liver stiffness measurement (LSM) by VCTE has been extensively studied among patients with CLD,^10^ and has been proposed as a noninvasive test for screening of GEV and portal hypertension.^11^, ^12^ Furthermore, it has been shown that spleen stiffness measurement (SSM) using the liver VCTE settings (SSM@50 Hz) could be used for noninvasive assessment, for monitoring of portal hypertension, and for detecting esophageal varices (EV).^13^ In order to overcome the limitations associated with the use of the liver VCTE settings for SSM, a novel, spleen‐dedicated examination based on VCTE has recently been developed. The FibroScan 630 Expert device is equipped with B‐mode ultrasound probe to help localize the spleen; it has spleen‐dedicated VCTE settings for the M probe with a fixed shear wave frequency at 100 Hz. It has adjusted measurement depth and stiffness range between 6 and 100 kPa. SSM using spleen mode (SSM@100 Hz) has been reported as a useful noninvasive test for screening of GEV in CLD.^14^, ^15^

The aim of this study was to directly compare the ability of LSM using liver VCTE settings, SSM using liver VCTE settings (SSM@50 Hz), and SSM using spleen VCTE settings (SSM@100 Hz) for the noninvasive assessment of GEV and portal hypertension.

## Methods

### Study design

This single‐center, cross‐sectional study complied with the Helsinki Declaration of 2013 and was approved by the Institutional Review Board of the Yokohama City University Hospital. It was registered in the Japan Registry of Clinical Trials (jRCTs032200119). Written informed consent was obtained from each enrolled subject.

### Patients

**Figure 1:**
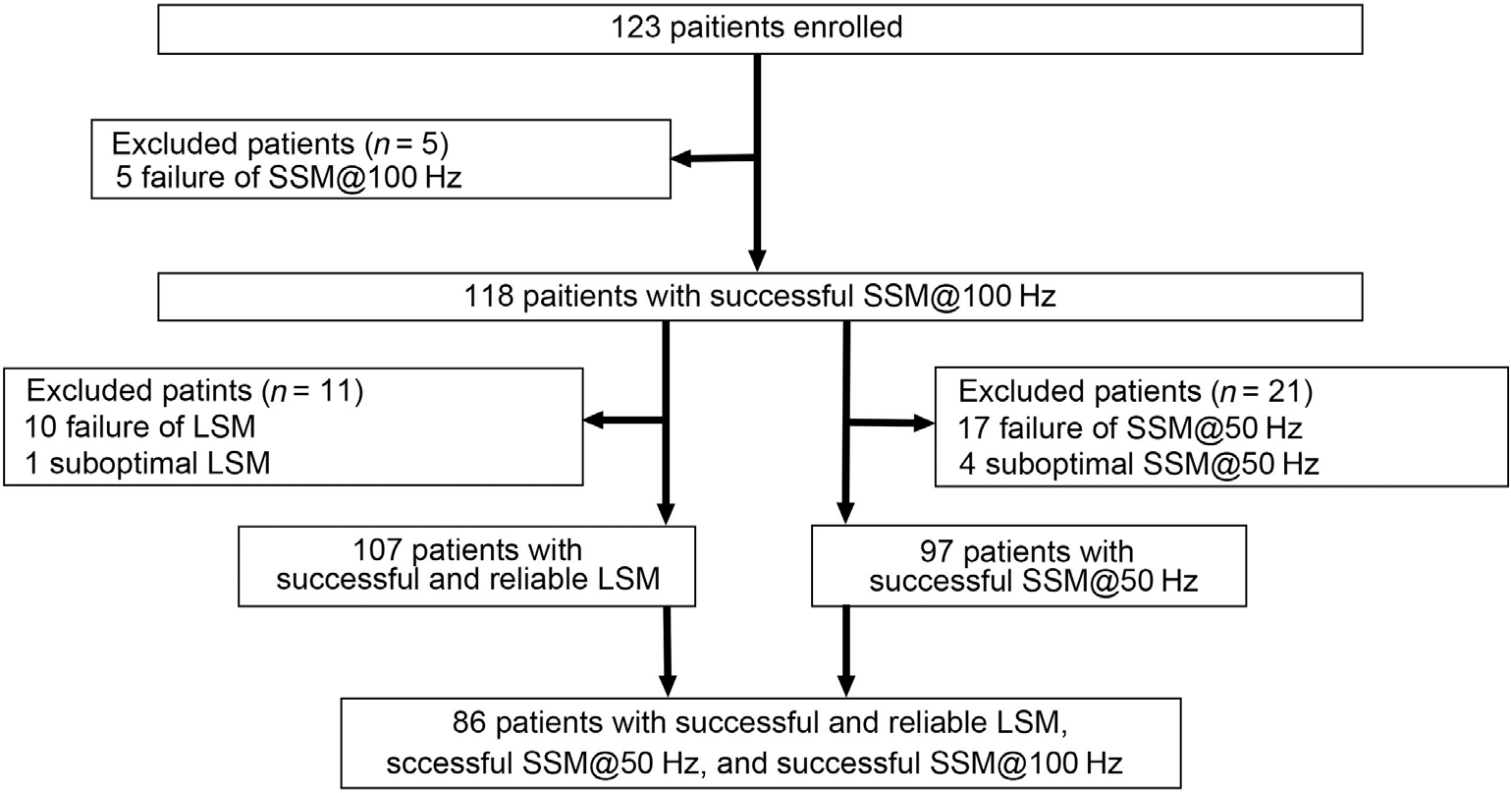
Patient flow scheme. LSM, liver stiffness measurement; SSM, spleen stiffness measurement.

This study was performed at the Yokohama City University Hospital, Yokohama, Japan. The study protocol is shown in Figure 1. A total of 123 patients with CLD were enrolled from October 2020 to May 2021. Inclusion criteria were: CLD due to nonalcoholic fatty liver disease (NAFLD), alcoholic liver disease (ALD), viral infections (hepatitis B virus [HBV] and hepatitis C virus [HCV]), or idiopathic portal hypertension (IPH), where patients were between 18 and 90 years old, and blood examination, EGD, and HVPG were performed within 12 weeks from VCTE. Venous blood was obtained conventionally in the morning following an overnight fast (12 h). Hepatocellular carcinoma (HCC) was diagnosed per international guidelines.^16^ Exclusion criteria included ascites around the spleen, pregnancy, use of a pacemaker, prior splenectomy, serum aminotransferases ≥250 IU/L, jaundice (defined by total serum bilirubin ≥10.0 mg/dL), and platelet count <10 000/μL.

There were no patients on selective β‐blockade (NSBB) or with portal vein tumors or thrombosis.

### Esophagogastroduodenoscopy

A standard EGD was performed. The EV^17^, ^18^ and gastric varices (GV)^19^ were graded I, II, and III, respectively, as follows in Supporting information. Presence of GEV is in the presence of any EV grade I and above and/or in presence of any GV grade I and above. High‐bleeding risk varices (HRV) were defined as grade II EV, grade III EV, or grade 1 EV with red signs, per the Baveno VI consensus.^20^, ^21^ In addition, Grade III GV were also defined as HRV. The main outcomes of interest were the presence of GEV and HRV.

### Liver stiffness measurement

LSM was assessed using standard liver VCTE settings of FibroScan 630 Expert (3.5‐MHz M and/or 2.5‐MHz XL probe, Echosens, Paris, France) after at least 12 h of fasting and under ultrasound guidance. Selection between the M or XL probe was based on the automatic probe selection tool embedded in the FibroScan software. All examinations were initiated with the M probe which was switched to the XL probe upon recommendation by the automatic probe selection tool. The examination was conducted by one expert experienced in both ultrasonography and VCTE. Reliable LSM was defined as LSM < 7 kPa or LSM ≥7.1 kPa with interquartile range (IQR) <30%.^22^ We excluded the LSM with less than 10 valid measurements. We defined failure of LSM as having no valid measurement at all and suboptimal LSM as having less than 10 valid measurements.

### Spleen stiffness measurement

SSM were performed using both the liver and spleen VCTE settings as described earlier^14^, ^15^ (FibroScan 630 Expert, Echosens) after at least 12 h of fasting and under ultrasound guidance. The patients were placed in a supine position with maximal abduction of the left arm, and the probe was positioned in an intercostal space where the spleen was correctly visualized by US. We only used the M probe regardless of skin capsule distance.

We defined failure of SSM as having no valid measurement at all and suboptimal SSM as having less than 10 valid measurements.

### Combination of noninvasive methods

The other noninvasive assessments were computed for each patient: aspartate aminotransferase and alanine aminotransferase ratio (AAR),^23^ aminotransferase to platelet ratio index (APRI),^24^ fibrosis‐4 (Fib‐4) index,^25^ platelet count to spleen diameter ratio (PSR),^26^ and LSM‐spleen diameter to platelet ratio score (LSPS).^27^ These scores are detailed in the Supporting information.

### Measurement of hepatic venous pressure gradient

A venous introducer was placed in the right internal jugular vein. The pressures in both the wedged and free positions were measured using a balloon‐tipped catheter. The HVPG was calculated by subtracting the free hepatic venous pressure from the wedged venous pressure. Measurement of HVPG was performed by one expert.

### Statistical analysis

Analysis of variance with Scheffe's multiple testing correction was used for univariate comparisons between groups. The Kruskal–Wallis test was used for comparisons of nonparametric data of more than two independent groups. Correlations between two variables were tested by calculating the Pearson correlation coefficient. The sensitivities and specificities for selected cut‐off values were determined, and the area under the receiver operating characteristics (AUROCs) were calculated. Cut‐off values were determined using an optimization step that maximized the Youden index. The *z*‐test was used for comparisons of the AUROC curve between two groups.^28^ Statistical analyses were performed using SPSS v12.0 (SPSS Inc., Chicago, IL, USA). All authors had access to the study data and reviewed and approved the final manuscript.

## Results

### Patient characteristics

Of 123 patients with CLD enrolled into the study, 90 patients (73.1%) had HCC. Five patients had SSM@100 Hz failure (2 with obesity [body mass index ≥ 30], 2 with a poorly delineated spleen, and 1 with a narrow intercostal space) (Fig. 1). Moreover, successful SSM@50 Hz could not be obtained for 21 patients, 17 patients had SSM@50 Hz failure (6 with obesity, 11 with a smaller longitudinal spleen diameter), and 4 patients had suboptimal studies. Valid LSM could not be obtained for 11 of the 118 patients with successful SSM@100 Hz where 10 patients had failure of LSM and one patient had suboptimal LSM. In summary, 97 patients successfully underwent both SSM@100 Hz and SSM@50 Hz, while 86 patients had successfully undergone SSM@100 Hz, SSM@50 Hz, and LSM studies.

Use of VCTE settings for the liver is suboptimal for SSM for several reasons. 100 Hz shear wave center frequency and measurement depths between 25 and 55 mm of SSM@100 Hz are suitable for SSM.

The characteristics of the 118 patients are shown in Table 1. The details of patients with GEV in this study are summarized in Table S1. Of 118 patients, 60 patients had varices and 20 patients were positive for the red color sign.

**Table 1:** Clinical and demographic characteristics in patients with chronic liver disease

	Non‐GEV		GEV		
	*n*	Mean ± SD	*n*	Mean ± SD	*P* value
Age (years)	58	71.3 ± 13.2	60	65.9 ± 12.3	0.023
Sex (female:male)	58	10:48	60	19:41	0.068
Body mass index (kg/m^2^)	58	25.0 ± 3.8	60	24.8 ± 4.9	0.770
SCD (mm)	58	18.2 ± 4.9	60	18.2 ± 4.6	0.962
HCV/HBV/alcohol/NAFLD/IPH/other	58	11/13/11/13/0/10	60	13/6/14/17/2/8	0.323
HCC (yes/no)	58	47/11	60	40/20	0.076
Platelet count (10^9^/L)	58	161 ± 73	60	111 ± 61	<0.001
PT (INR)	58	1.10 ± 0.11	60	1.21 ± 0.20	<0.001
Albumin (g/dL)	58	3.9 ± 0.5	60	3.4 ± 0.6	<0.001
Total bilirubin (mg/dL)	58	0.9 ± 0.7	60	1.4 ± 1.1	0.007
AST (U/L)	58	40.7 ± 30.6	60	45.2 ± 31.6	0.439
ALT (U/L)	58	31.2 ± 19.2	60	30.6 ± 27.1	0.892
Child‐Pugh classification (A/B/C)	58	50/7/1	60	38/18/4	0.016
Spleen diameter (cm)	58	9.7 ± 2.1	60	12.3 ± 2.6	<0.001
LSM 50 Hz (kPa)	55	16.6 ± 13.9	52	30.0 ± 17.8	<0.001
SSM@50 Hz (kPa)	47	22.5 ± 14.3	50	48.2 ± 18.5	<0.001
SSM@100 Hz (kPa)	58	24.5 ± 10.1	60	57.1 ± 18.9	<0.001
HVPG (mmHg)	5	4.2 ± 1.6	15	10.5 ± 5.4	0.021
GEV (EV/GV/EV + GV)	58	0/0/0	60	42/4/14	<0.001

ALT, alanine aminotransferase; AST, aspartate aminotransferase; EV, esophageal varices; GEV, gastroesophageal varices; GV, gastric varices; HBV, hepatitis B virus; HCC, hepatocellular carcinoma; HCV, hepatitis C virus; HVPG, hepatic venous pressure gradient; INR, international normalized ratio; IPH, idiopathic portal hypertension; LSM, liver stiffness measurement; NAFLD, non‐alcoholic fatty liver disease; PT, prothrombin time; SCD, skin capsule distance; SSM_50_, spleen stiffness measurement with liver mode; SSM_100,_ spleen stiffness measurement with spleen mode.

### Diagnostic accuracy for gastroesophageal varices

**Figure 2:**
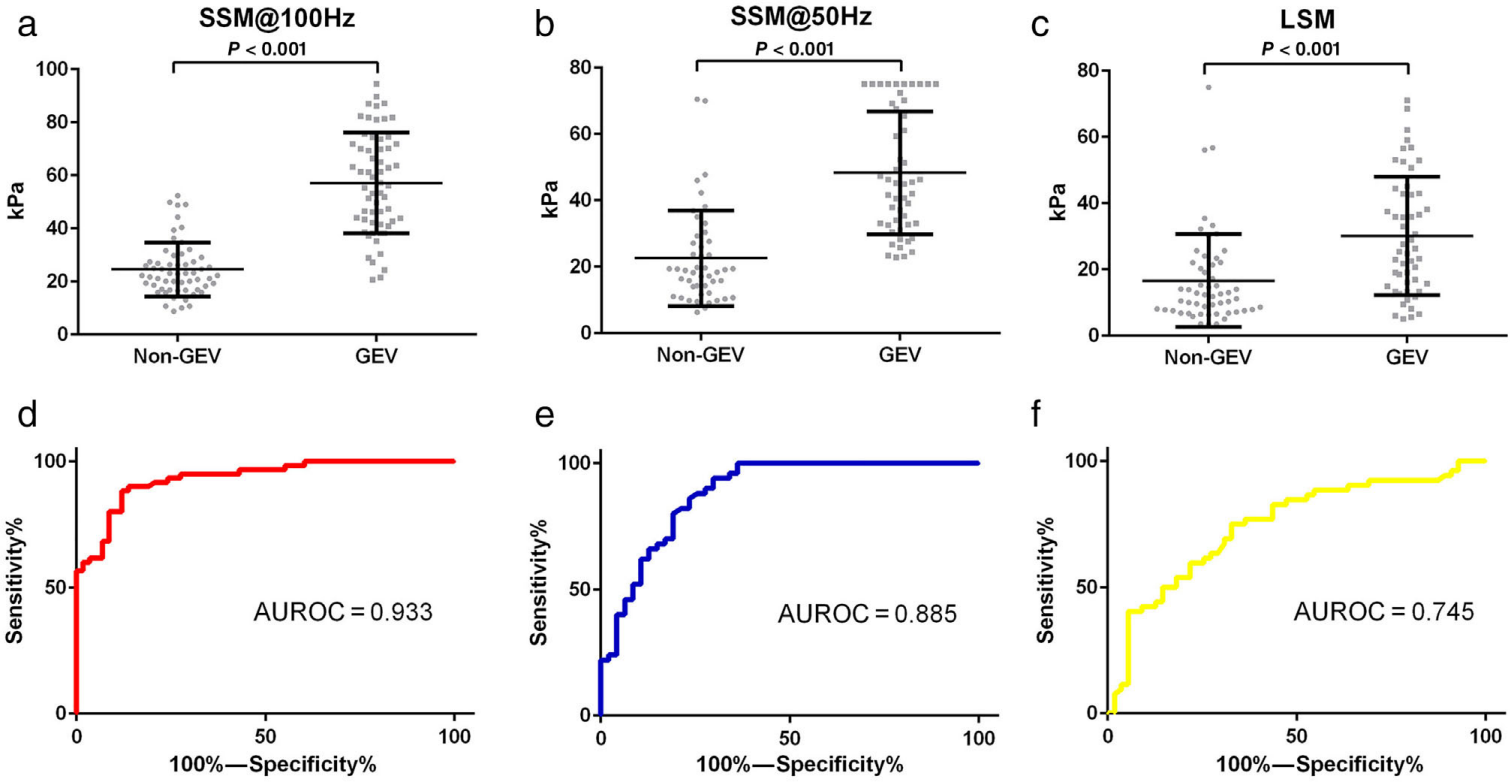
Distribution and receiver operating characteristic (ROC) curves for spleen stiffness measurement (SSM)@100 Hz, SSM@50 Hz, and liver stiffness measurement (LSM) among chronic liver disease patients with and without gastroesophageal varices (GEV). Distribution of (a) SSM@100 Hz, (b) SSM@50 Hz, and (c) LSM among patients with and without GEV. ROC curves for (d) SSM@100 Hz, (e) SSM@50 Hz, and (f) LSM for identifying GEV. AUROC, area under the ROC.

The mean values of SSM@100 Hz, SSM@50 Hz, and LSM in the patients with GEV were significantly higher than those of patients with non‐GEV, respectively (Table 1). Among patients with 10 successful valid measurements, AUROC, sensitivity, specificity, positive predictive value, and negative predictive value for the diagnosis of GEV with SSM@100 Hz, SSM@50 Hz, LSM, and other non‐invasive tests (NITs) (AAR, APRI, Fib‐4 index, PSR, LSPS, platelet count) are presented in Table S2 and Figure 2. SSM@100 Hz was the best tool for the diagnosis of GEV in patients with CLD (AUROC = 0.933). However, the number of the patients in each VCTE was different.

We then analyzed 86 patients who successfully underwent SSM@100 Hz, SSM@50 Hz, and LSM. SSM@100 Hz was shown to be the best tool for the diagnosis of GEV among patients with CLD (AUROC = 0.944) (Figure S1, Table S3). The AUROC of SSM@100 Hz and of SSM@50 Hz for diagnosing GEV were significantly higher than that of LSM. LSPS was the best (AUROC = 0.841) among the evaluated noninvasive scores; however, the AUROC of LSPS was significantly lower than that of SSM@100 Hz (*P* = 0.005).

Among those with liver cirrhosis, defined as LSM ≥11.8 kPa,^29^ we analyzed 55 patients who successfully underwent SSM@100 Hz, SSM@50 Hz, and LSM. Figure S2 and Table S4 show direct comparisons of the diagnostic accuracy between SSM@100 Hz and SSM@50 Hz, LSM, and other NITs. The AUROC of SSM@100 Hz for the diagnosis of GEV was the highest (0.916) and that was significantly higher than that of LSM in detecting GEV.

### Direct comparison of SSM@100 Hz and SSM@50 Hz for the diagnostic accuracy of GEV

Subsequently, we analyzed the 97 patients with successful SSM@100 Hz and SSM@50 Hz to compare their accuracy for diagnosing GEV. Figure S3 and Table S5 show direct comparisons of the diagnostic accuracy of SSM@100 Hz, SSM@50 Hz, and other NITs. SSM@100 Hz was correlated with SSM@50 Hz (*n* = 97, *r* = 0.834, *P* < 0.001) (Figure S4). SSM@100 Hz was the best tool for the diagnosis of GEV in the patients with CLD (AUROC = 0.918). However, there were no significant differences between the AUROC of SSM@100 Hz and SSM@50 Hz for detecting GEV (Table S5).

The values of SSM@50 Hz and SSM@100 Hz for EV and GV subgroups (grade I, II, III) in the patients with CLD were analyzed (Figure S5). Among patients with EV, the median values of SSM@100 Hz and SSM@50 Hz were significantly different, respectively (Kruskal–Wallis test, *P* < 0.001). In particular, a steady stepwise increase of SSM@100 Hz was observed with increasing severity and grade of EV. Among those with EV and GV of grade ≥ I (*n* = 50), the median values for SSM@100 Hz of among those GEV who were also positive for red color sign (RC+) were significantly higher than those with GEV who negative for red color sign (RC−) (*P* < 0.001) (Figure S6). On the other hand, the median values for SSM@50 Hz did not show significant difference between those with GEV who were RC+ and those with GEV who were RC− (*P* = 0.101).

**Figure 3:**
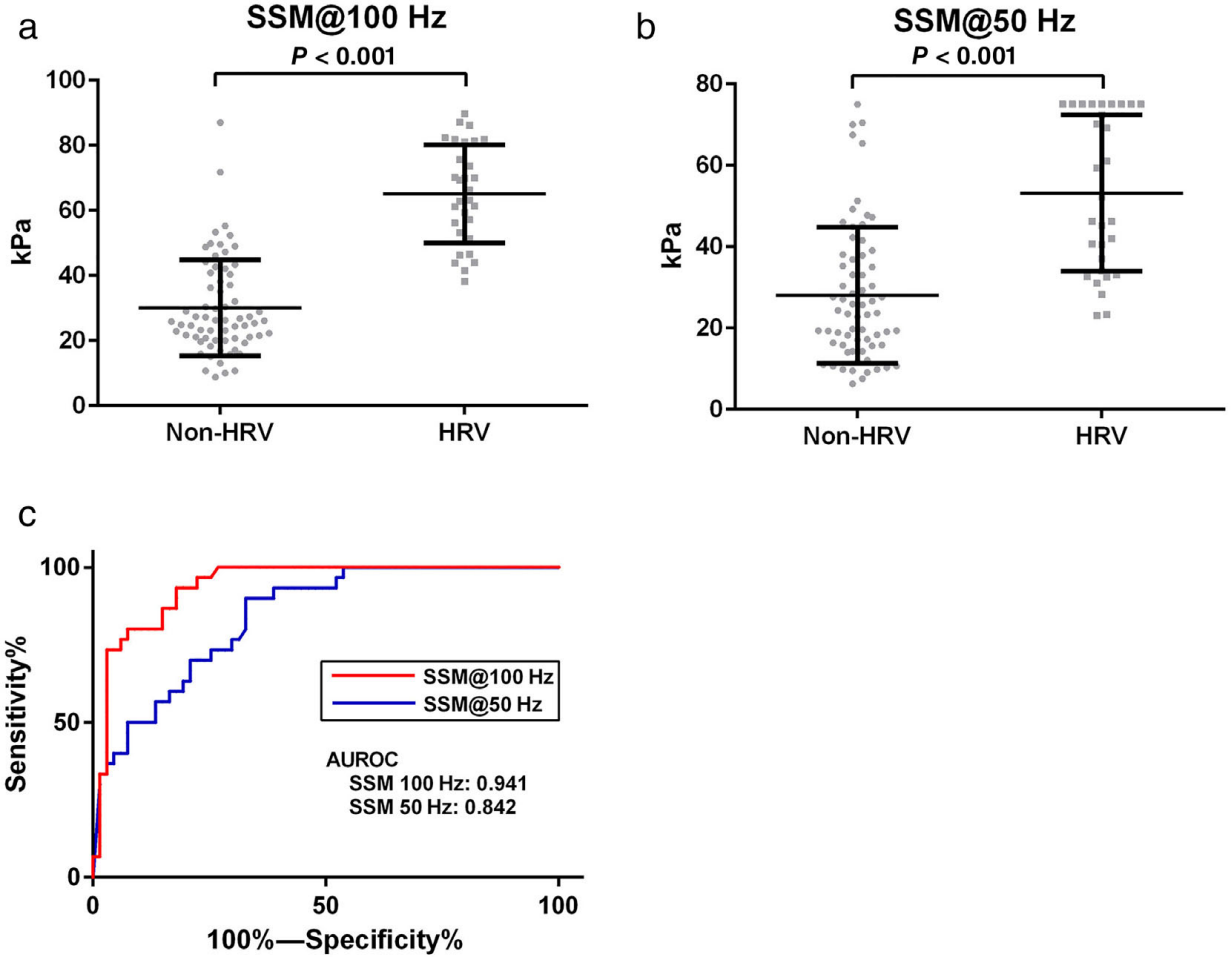
Distribution and direct comparison of receiver operating characteristic (ROC) curves of spleen stiffness measurement (SSM)@100 Hz and SSM@50 Hz for identifying high‐bleeding risk varices (HRV) in patients with chronic liver disease (CLD). Distribution of (a) SSM@100 Hz and (b) SSM@50 Hz among CLD patients with and without HRV. (c) Direct comparison of ROC curves of SSM@100 Hz and SSM@50 Hz for identifying HRV. AUROC, area under the ROC.

HRV always requires the attention of physicians; protection from variceal hemorrhage is paramount in the management of patients with advanced CLD. In Figure 3, the mean values of SSM@100 Hz and SSM@50 Hz among patients with HRV (*n* = 30) were 65.0 ± 15.0 kPa and 53.1 ± 19.2 kPa, which were significantly higher than those of patients without HRV (*n* = 67) (30.1 ± 14.7 kPa, *P* < 0.001; 28.0 ± 16.7 kPa, *P* < 0.001), respectively. Furthermore, the AUROC of SSM@100 Hz for the diagnosis of HRV was 0.941, which was significantly higher than that of SSM@50 Hz (AUROC = 0.842) (*P* = 0.002) (Fig. 3C, Table 2). The AUROC of SSM@50 Hz for detecting HRV was better for the controlled attenuation parameter (CAP) >118 dB/m group (0.940) than for the CAP ≤118 dB/m group (0.801) (Table S6).

**Table 2:** Direct comparisons of the diagnostic accuracy of SSM@100 Hz, SSM@50 Hz, and other noninvasive tests in detecting HRV using esophagogastroduodenoscopy as the reference in the subgroup of patients with 10 valid measurements for all VCTE procedures

	*n*	AUROC	95% CI	*P* value^†^	*Versus* SSM@50 Hz *P* value	*Versus* SSM@100 Hz *P* value	Cut‐off level	Sensitivity	Specificity	PPV	NPV
Elastography											
SSM@100 Hz	97	0.941	0.896–0.986	<0.001	0.002	—	43.8	93.3	82.0	70.0	96.4
SSM@50 Hz	97	0.842	0.764–0.919	<0.001	—	0.002	31.0	90.0	67.1	55.1	93.7
Scoring systems											
AAR	97	0.653	0.540–0.765	0.016	0.006	<0.001	1.29	80.0	50.7	42.1	85.0
APRI	97	0.800	0.706–0.893	<0.001	0.426	0.002	0.844	83.3	73.1	58.1	90.7
Fib‐4 index	97	0.824	0.736–0.913	<0.001	0.750	0.013	4.65	86.6	70.1	56.5	92.1
PSR	97	0.769	0.671–0.868	<0.001	0.143	<0.001	1063.6	86.6	67.1	54.1	91.8
Single marker											
Platelet count	97	0.740	0.634–0.847	<0.001	0.068	<0.001	123	80.0	65.6	51.0	88.0

AAR, AST to ALT ratio; APRI, AST to platelet ratio index.

AAR, alanine aminotransferase ratio; APRI, aminotransferase to platelet ratio index; AUROC, area under the receiver operating characteristic; CI, confidence interval; Fib‐4, fibrosis‐4 score; GEV, gastroesophageal varices; HRV, high‐bleeding risk varices; NPV, negative predictive value; PPV, positive predictive value; PSR, platelet count to spleen diameter ratio; SSM@50 Hz, spleen stiffness measurement with liver mode; SSM@100 Hz_,_ spleen stiffness measurement with spleen mode; vibration‐controlled transient elastography; VCTE, vibration‐controlled transient elastography.

^†^ Presence of GEV *versus* non‐GEV.

### Spleen stiffness measurement comparison with hepatic venous pressure gradient

Considering that HVPG and LSM values in IPH are much lower than the cut‐off for CSPH in cirrhosis,^30^ we excluded such patients in this analysis of HVPG. HVPG was measured in 18 patients. Table S7 shows the characteristics of the patients who underwent HVPG. The prevalence of CSPH was 50.0%. On univariate analysis, patients with CSPH had a higher AST, SSM@50 Hz, and SSM@100 Hz as compared with those who did not have CSPH (Table S7).

**Figure 4:**
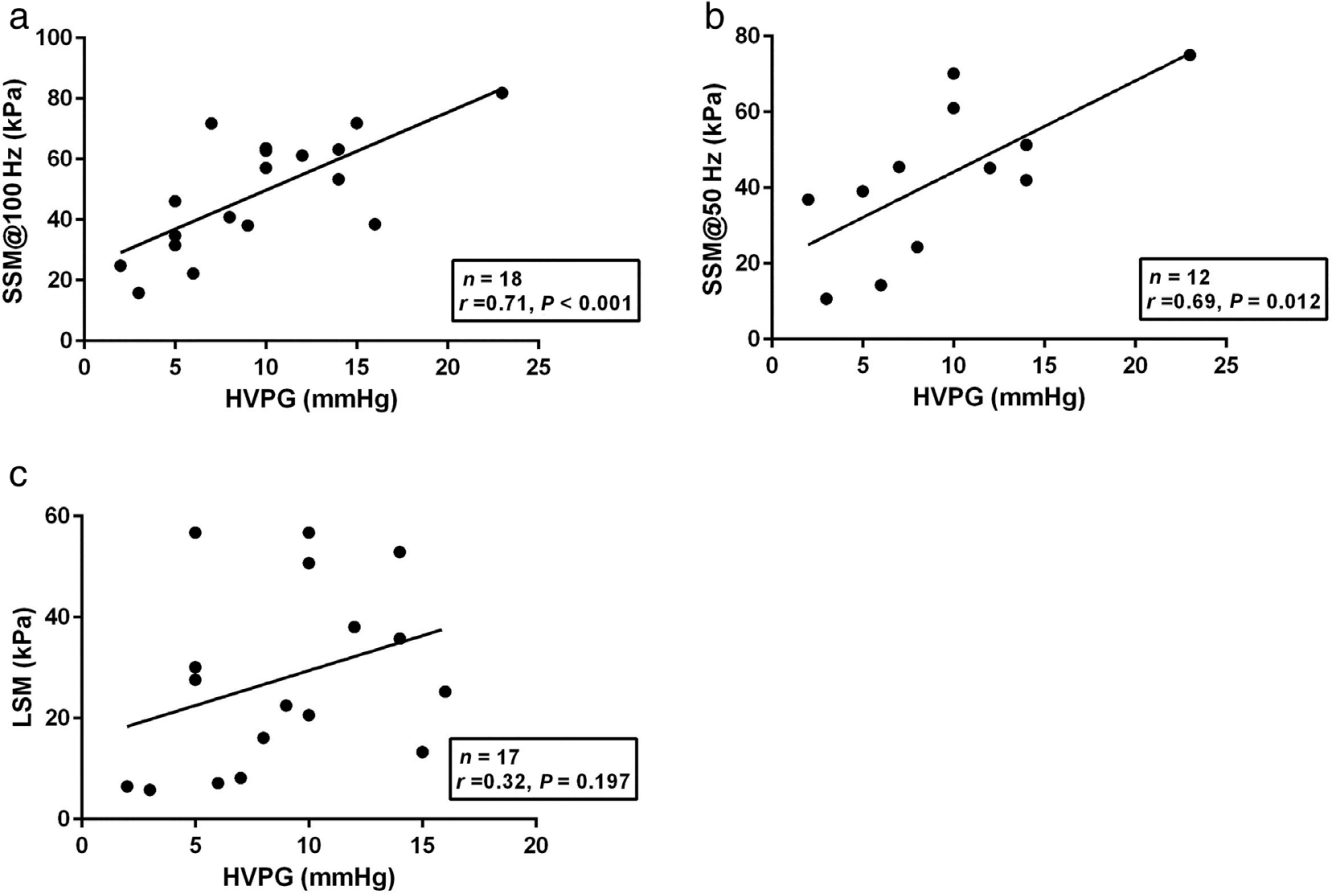
Linear regression analysis of the correlation between hepatic venous pressure gradient (HVPG) and spleen stiffness measurement (SSM)@100 Hz, SSM@50 Hz, and liver stiffness measurement (LSM) in patients with chronic liver disease. Correlation between HVPG (mmHg) and (a) SSM@100 Hz (kPa) (*n* = 18, *r* = 0.71, *P* < 0.001), (b) SSM@50 Hz (kPa) (*n* = 12, *r* = 0.69, *P* = 0.012) and (c) LSM (kPa) (*n* = 17, *r* = 0.32, *P* = 0.197).

HVPG was significantly higher among patients with GEV (11.7 ± 4.6 mmHg, *n* = 13) than in those without (4.2 ± 1.6 mmHg, *n* = 5) (*P* = 0.003). Furthermore, HVPG was better correlated with SSM@100 Hz (*n* = 18, *r* = 0.71, *P* < 0.001) than LSM (*n* = 17, *r* = 0.32, *P* = 0.197) and SSM@50 Hz (*n* = 12, *r* = 0.69, *P* = 0.012) (Fig. 4).

## Discussion

In this cross‐sectional study, we demonstrated that SSM@100 Hz, SSM@50 Hz, and LSM are useful NITs for the diagnosis of GEV in the patients with CLD. The cases of patients with failure and suboptimal spleen stiffness measurements were lower in SSM@100 Hz (5 out of 123, 4.0%) than in SSM@50 Hz (21 out of 118, 17.7%). Moreover, SSM@100 Hz remained below the predefined range upper value 100 kPa, while SSM@50 Hz reached the predefined range upper value (75 kPa) in 10 patients. The AUROC of SSM@100 Hz for the diagnosis of HRV was significantly higher (0.941) than that of SSM@50 Hz (0.842).

As previously reported, we also showed that SSM@50 Hz and SSM@100 Hz were useful for the diagnosis of GEV.^13^, ^14^, ^15^ Our study showed that the AUROC of SSM@100 Hz and SSM@50 Hz were significantly higher than that of LSM for the diagnosis of GEV; moreover, SSM@100 Hz had very high diagnostic accuracy in detecting GEV (AUROC = 0.933). Stefanescu demonstrated that AUROC for HRV with SSM@100 Hz was significantly higher than that of LSM.^15^ Our results not only echoed the same but also showed that AUROC for HRV, with SSM@100 Hz, was significantly higher than that of SSM@50 Hz; moreover, the AUROC measured in this study was higher than that of the previous report. SSM@100 Hz showed higher specificity (82.0%) than SSM@50 Hz (67.1%) for the diagnosis of HRV, which means that SSM@100 Hz would more efficiently diagnose varices which require treatment. Our result showed that SSM@100 Hz showed higher AUROC for the diagnosis of GEV and HRV than that of Stefanescu's study which used the same novel spleen‐dedicated VCTE (SSM@100 Hz).

Hirooka *et al*. showed that SSM@50 Hz was significantly correlated with HVPG (*n* = 148; *r* = 0.558).^31^ In our study, we showed that SSM@50 Hz was significantly correlated with HVPG (*n* = 12; *r* = 0.69) and that SSM@100 Hz was significantly correlated with HVPG (*n* = 18; *r* = 0.71). Although SSM@50 Hz and SSM@100 Hz was more highly correlated with HVPG in our study, our study had fewer patients than the study by Hirooka *et al*. Stefanescu showed correlations between HVPG and SSM@50 Hz (*n* = 102; *r* = 0.363) and between HVPG and SSM@100 Hz (*n* = 102; *r* = 0.532).^15^ Further studies are needed to assess the correlations between HVPG and SSM@100 Hz. Hirooka *et al*. also showed that the AUROC of SSM@50 Hz for detecting HRV was better for the low CAP group (CAP ≤118 dB/m) than for the high CAP group (CAP > 118 dB/m).^31^ Our results were in contrast to theirs. However, there were fewer patients in our study, especially in high CAP group. Therefore, further studies are needed.

An effective screening tool should be noninvasive, fast, easily accessible, and cost‐effective, for prompt diagnosis and for prevention of life‐threatening adverse effects and resulting mortality. Alternatives to EGD have been investigated. However clinical scoring systems (AAR, APRI, Fib‐4‐index, PSR, and LSPS) did not show satisfactory all‐around diagnostic performance in our study. Previous studies have indicated that LSM can be used to diagnose GEV in patients with CLD.^9^, ^21^ Based on the recent 2015 Baveno VI consensus workshop, the combination of LSM assessed by VCTE and platelet count was useful for the diagnosis of HRV, allowing for safe avoidance of EGD.^21^ Nevertheless, LSM has a poor correlation with portal pressure and its resulting complications as HVPG rises above 10 mmHg. Liver stiffness cannot reflect the complex hemodynamic changes characteristic of hyperdynamic syndrome and the opening of portosystemic shunts.^32^ Accordingly, LSM might underestimate the severity of PH and the risk of variceal bleeding.

Use of VCTE settings for the liver is suboptimal for SSM for several reasons: First, it can be challenging to localize the spleen without B‐mode ultrasound; second, when the spleen is not enlarged the measurement depths for the liver settings are set between 25 and 65 mm, which can be too large; and third, the range of stiffness of the liver between 1.5 and 75 kPa are too narrow since spleen is stiffer than the liver. The spleen‐dedicated stiffness measurement feature now available on the FibroScan 630 Expert device includes an embedded B‐mode scanner for better localization of the spleen with VCTE settings adapted to the peculiarities of the spleen. These include operating at 100 Hz shear wave center frequency to avoid overestimation, measurement depths between 25 and 55 mm below the probe, and measurement range between 5 and 100 kPa. Considering these, our study suggests that SSM@100 Hz is clinically reliable. It is recommended that LSM should be periodically repeated for patients with CLD.^10^, ^33^ We propose performing both SSM@100 Hz/SSM@50 Hz and LSM at the same time.

Of note, patients with IPH frequently have hepatic vein‐to‐vein communications and, despite unequivocal signs of portal hypertension, their HVPG and LSM values are much lower than the cut‐off for CSPH in cirrhosis.^30^ Despite this, SSM in this population demonstrated high values similar to or even higher than those observed in patients with cirrhotic PH.^34^ In our study, two patients with IPH were enrolled and their HVPG and LSM values were low; however, their SSM@100 Hz values were high and they correspondingly had GEV.

Portopulmonary hypertension (PoPH) is a severe complication of CLD.^35^, ^36^ PoPH is also a devastating complication of portal hypertension.^37^, ^38^ PoPH has a very poor prognosis; therefore, useful noninvasive tests for PoPH are needed. We did not analyze PoPH with SSM during this study. It is needed to assess whether SSM is useful for predicting the severity of PoPH.

This study had several limitations. First, there is no validation population in this study. As this study was performed in a single‐center within a short period of time, a validation population was not set up. Second, the ethnicity of the subjects was homogeneous, in that all the patients were Japanese. Therefore, a similar study to confirm the findings among subjects of various ethnicities is needed. Third, this was a cross‐sectional study, and further studies are needed to assess the changes longitudinally. Fourth, few patients had HVPG measurement. More patients with simultaneous comparison between HVPG and SSM@100 Hz, would be needed to confirm our results.

In conclusion, SSM@100 Hz has higher diagnostic accuracy in detecting HRV than other NITs. SSM@100 Hz will greatly help allocate interventions among CLD patients from different categories of risk and guide further evaluation. SSM@100 Hz can efficiently differentiate patients with GEV and HRV from patients with CLD. Therefore, the use EGD for determining the definitive diagnosis of GEV and HRV will decrease. SSM@100 Hz is noninvasive and reduces costs compared to EGD. Further investigation is required to evaluate the prognostic value of SSM@100 Hz for long‐term outcomes and post‐treatment evaluation and prediction of relapse in HRV.

## Supporting information


**Appendix** **S1.** Supporting information.
