# Supplementary material for: Gastroesophageal varices evaluation using spleen‐dedicated stiffness measurement by vibration‐controlled transient elastography
Source: JGH Open. 2021 Dec 14;6(1):11–9. doi: 10.1002/jgh3.12689 (PMC8762624; doi:10.1002/jgh3.12689)
Supplement: Supplementary file 1 — Appendix S1. Supporting information. [file JGH3-6-11-s001.docx]

**Supplementary Material**

***Supplementary Methods***

*Esophagogastroduodenoscopy*

Esophagogastroduodenoscopy (EGD) was performed by an endoscopist, findings were accordingly recorded. The esophageal varices (EV) were graded as follows^1, 2^: grade I, EV were flattened by insufflation; grade II, EV were nonconfluent and protruded into the lumen despite insufflation; grade III, EV were confluent and were not flattened by insufflation. The gastric varices (GV) were graded as follows based on form^3^: grade I, GV were straight, small-caliber varices; grade II, GV were moderately enlarged, beady varices; grade III, GV were markedly enlarged, nodular or tumor-shaped varices. The presence of red signs and gastric varices was also recorded in all patients. At the end of the study, all endoscope images were independently and blindly evaluated by two experts; the resulting consensus was used for the analysis.

*Combination of noninvasive methods*

AAR ^4^: AST/AST

APRI ^5^: [AST / ULN] / [platelet counts (10^9^/L)] x 100

Fib-4 ^6^: [age × AST] / [platelet count (10^9^/L) × √ALT]

Platelet count to spleen diameter ratio (PSR): platelet count (n/mm^3^) / bipolar diameter (mm) of the spleen ^7^.

LSM-spleen diameter to platelet ratio score (LSPS): [liver stiffness measurement (LSM) × spleen diameter] / [platelet count (10^9^/L)] ^8^.

**Supplementary Reference**

1. Calès P, Buscail L, Bretagne JF, et al. [Interobserver and intercenter agreement of gastro-esophageal endoscopic signs in cirrhosis. Results of a prospective multicenter study]. Gastroenterol Clin Biol 1989; 13:967-973.
2. Calès P, Zabotto B, Meskens C, et al. Gastroesophageal endoscopic features in cirrhosis. Observer variability, interassociations, and relationship to hepatic dysfunction. Gastroenterology 1990; 98:156-162.
3. Tajiri T, Yoshida H, Obara K, et al. General rules for recording endoscopic findings of esophagogastric varices (2nd edition). Dig Endosc 2010; 22:1-9.
4. Giannini E, Risso D, Botta F, et al. Validity and clinical utility of the aspartate aminotransferase-alanine aminotransferase ratio in assessing disease severity and prognosis in patients with hepatitis C virus-related chronic liver disease. Arch Intern Med 2003; 163:218-224.
5. Wai CT, Greenson JK, Fontana RJ, et al. A simple noninvasive index can predict both significant fibrosis and cirrhosis in patients with chronic hepatitis C. Hepatology 2003; 38:518-526.
6. Sterling RK, Lissen E, Clumeck N, et al. Development of a simple noninvasive index to predict significant fibrosis in patients with HIV/HCV coinfection. Hepatology 2006; 43:1317-1325.
7. Giannini E, Botta F, Borro P, et al. Platelet count/spleen diameter ratio: proposal and validation of a non-invasive parameter to predict the presence of oesophageal varices in patients with liver cirrhosis. Gut 2003; 52:1200-1205.
8. Kim BK, Han KH, Park JY, et al. A liver stiffness measurement-based, noninvasive prediction model for high-risk esophageal varices in B-viral liver cirrhosis. Am J Gastroenterol 2010; 105:1382-1390.

**Supplementary Table 1.** Characteristics of GEV in patients with CLD

| N=60 |  | N (%) |
| --- | --- | --- |
| EV |  | 42 (70.0) |
| GV |  | 4 (6.7) |
| EV + GV |  | 14 (23.3) |
| Grade of EV |  |  |
| Grade 0 |  | 4 (6.7) |
| Grade 1 |  | 25 (41.7) |
| Grade 2 |  | 20 (33.3) |
| Grade 3 |  | 11 (18.3) |
| Red color signs of EV |  |  |
| Red wale marks |  | 2 (3.3) |
| Cherry spots |  | 16 (26.7) |
| Hematocystic spot |  | 2 (3.3) |
| Grade of GV |  |  |
| Grade 0 |  | 0 (0) |
| Grade 1 |  | 8 (13.3) |
| Grade 2 |  | 7 (11.7) |
| Grade 3 |  | 3 (5.0) |
| Red color signs of GV |  |  |
| Red wale marks |  | 0 (0) |
| Cherry spots |  | 0 (0) |
| Hematocystic spot |  | 0 (0) |

EV, esophageal varices; GEV, gastroesophageal varices; GV, gastric varices; CLD, chronic liver disease

**Supplementary Table 2.** Comparisons of the diagnostic accuracy between SSM@100Hz, SSM@50Hz, LSM, and other non-invasive tests in detecting GEV using EGD as the reference, among patients with 10 valid measurements for each VCTE procedure

|  | N | AUROC | 95% CI | P value* | Cut-off level | Sensitivity | Specificity | PPV | NPV |
| --- | --- | --- | --- | --- | --- | --- | --- | --- | --- |
| **Elastography** | | | | | | | | | |
| SSM@100Hz | 118 | 0.933 | 0.891-0.975 | <0.001 | 37.1 | 88.3 | 87.9 | 88.3 | 87.9 |
| SSM@50Hz | 97 | 0.885 | 0.818-0.952 | <0.001 | 24.4 | 87.5 | 77.0 | 79.6 | 86.0 |
| LSM | 107 | 0.745 | 0.650-0.840 | <0.001 | 15.7 | 75.0 | 67.2 | 68.4 | 74.0 |
| **Scoring systems** | | | | | | | | | |
| AAR | 118 | 0.660 | 0.561-0.760 | 0.002 | 1.38 | 66.6 | 65.5 | 66.6 | 65.5 |
| APRI | 118 | 0.699 | 0.604-0.794 | <0.001 | 0.84 | 61.6 | 74.1 | 71.1 | 65.1 |
| Fib-4 index | 118 | 0.714 | 0.622-0.806 | <0.001 | 4.55 | 68.3 | 70.6 | 70.6 | 68.3 |
| PSR | 118 | 0.801 | 0.720-0.882 | <0.001 | 983.3 | 70.0 | 84.4 | 82.3 | 73.1 |
| LSPS | 107 | 0.838 | 0.764-0.913 | <0.001 | 1.15 | 90.3 | 65.4 | 71.2 | 87.8 |
| **Single marker** | | | | | | | | | |
| Platelet count | 118 | 0.750 | 0.661-0.840 | <0.001 | 12.3 | 71.6 | 72.4 | 72.8 | 71.1 |

Abbreviations: AAR, AST to ALT ratio; APRI, AST to platelets ratio index; CI, confidence interval; EGD, esophagogastroduodenoscopy; EV, esophageal varices; Fib-4, Fibrosis-4; GEV, gastroesophageal varices; GV, gastric varices; HVPG, hepatic venous pressure gradient; LSM, liver stiffness measurement; LSPS, LSM-spleen diameter to platelet ratio score; PSR, platelet count to spleen diameter ratio; SSM@50Hz, spleen stiffness measurement with liver mode; SSM@100Hz_,_ spleen stiffness measurement with spleen mode; VCTE, vibration controlled transient elastography

* Presence of GEV vs non-GEV

**Supplementary Table 3.** Direct comparisons of the diagnostic accuracy of SSM@100Hz, SSM@50Hz, LSM, and non-invasive tests in detecting GEV using EGD as the reference in patients with 10 valid measurements for all VCTE procedures.

|  | N | AUROC | 95% CI | P value* | vs LSM  P value | vs SSM@50Hz  P value | vs SSM@100Hz  P value | Cut-off level | Sensitivity | Specificity | PPV | NPV |
| --- | --- | --- | --- | --- | --- | --- | --- | --- | --- | --- | --- | --- |
| **Elastography** |  | | | | | | | | | | | |
| SSM@100Hz | 86 | 0.944 | 0.899-0.989 | < 0.001 | < 0.001 | 0.119 | - | 35.1 | 92.8 | 86.3 | 86.6 | 92.6 |
| SSM@50Hz | 86 | 0.899 | 0.832-0.966 | < 0.001 | 0.018 | - | 0.119 | 24.4 | 95.2 | 72.7 | 76.9 | 94.1 |
| LSM | 86 | 0.760 | 0.655-0.864 | < 0.001 | - | 0.018 | <0.001 | 15.7 | 73.8 | 75.0 | 73.8 | 75.0 |
| **Scoring systems** | | | | | | | | | | | | |
| AAR | 86 | 0.689 | 0.575-0.803 | 0.002 | 0.301 | 0.003 | <0.001 | 1.29 | 76.1 | 63.6 | 66.6 | 73.6 |
| APRI | 86 | 0.694 | 0.581-0.807 | 0.001 | 0.326 | <0.001 | <0.001 | 0.844 | 61.9 | 75.0 | 70.2 | 67.3 |
| Fib-4 index | 86 | 0.720 | 0.612-0.828 | < 0.001 | 0.542 | 0.003 | <0.001 | 6.01 | 47.6 | 90.9 | 83.3 | 64.5 |
| PSR | 86 | 0.806 | 0.713-0.899 | < 0.001 | 0.511 | 0.044 | 0.001 | 1063.6 | 73.8 | 79.5 | 77.5 | 76.0 |
| LSPS | 86 | 0.841 | 0.758-0.923 | < 0.001 | 0.025 | 0.163 | 0.005 | 1.15 | 90.4 | 68.1 | 73.0 | 88.2 |
| **Single marker** |  | | | | | | | | | | | |
| Platelet counts | 86 | 0.745 | 0.638-0.852 | < 0.001 | 0.850 | 0.006 | <0.001 | 10.3 | 61.9 | 81.8 | 76.4 | 69.2 |

Abbreviations: AAR, AST to ALT ratio; APRI, AST to platelets ratio index; CI, confidence interval; EGD, esophagogastroduodenoscopy; Fib-4, Fibrosis-4; HVPG, hepatic venous pressure gradient; LSM, liver stiffness measurement; LSPS, LSM x spleen diameter to platelet ratio score; PSR, platelet count to spleen diameter ratio; SSM@50Hz, spleen stiffness measurement with liver mode; SSM@100Hz, spleen stiffness measurement with spleen mode; VCTE, vibration controlled transient elastography

* Presence of GEV vs non-GEV

**Supplementary Table 4.** Dir0ect comparisons of the diagnostic accuracy of SSM@100Hz, SSM@50Hz, LSM, and non-invasive tests in detecting GEV using EGD as the reference in patients with 10 valid measurements for all VCTE procedures in patients with cirrhosis

|  | N | AUROC | 95% CI | P value* | vs LSM  P value | vs SSM@50Hz  P value | vs SSM@100Hz  P value | Cut-off level | Sensitivity | Specificity | PPV | NPV |
| --- | --- | --- | --- | --- | --- | --- | --- | --- | --- | --- | --- | --- |
| **Elastgraphy** |  | | | | | | | | | | | |
| SSM@100Hz | 55 | 0.916 | 0.843-0.990 | < 0.001 | 0.021 | 0.099 | - | 37.1 | 94.1 | 80.9 | 88.8 | 89.4 |
| SSM@50Hz | 55 | 0.834 | 0.719-0.950 | < 0.001 | 0.332 | - | 0.099 | 28.2 | 91.1 | 66.6 | 81.5 | 82.3 |
| LSM | 55 | 0.738 | 0.597-0.879 | 0.003 | - | 0.332 | 0.021 | 35.8 | 52.9 | 90.4 | 90.0 | 54.2 |
| **Scoring systems** | | | | | | | | | | | | |
| AAR | 55 | 0.718 | 0.567-0.869 | 0.006 | 0.816 | 0.284 | 0.039 | 1.29 | 82.3 | 61.9 | 77.7 | 68.4 |
| APRI | 55 | 0.583 | 0.433-0.733 | 0.302 | 0.115 | 0.005 | <0.001 | 1.278 | 50.0 | 76.1 | 77.2 | 48.4 |
| Fib-4 index | 55 | 0.642 | 0.498-0.787 | 0.077 | 0.341 | 0.034 | <0.001 | 6.05 | 50.0 | 85.7 | 85.0 | 51.4 |
| PSR | 55 | 0.694 | 0.554-0.835 | 0.016 | 0.695 | 0.049 | <0.001 | 753.9 | 50.0 | 90.4 | 89.4 | 52.7 |
| LSPS | 55 | 0.782 | 0.659-0.906 | 0.0005 | 0.516 | 0.458 | 0.018 | 1.66 | 85.2 | 61.9 | 78.3 | 72.2 |
| **Single marker** |  | | | | | | | | | | | |
| Platelet counts | 55 | 0.633 | 0.486-0.781 | 0.098 | 0.359 | 0.014 | <0.001 | 7.7 | 38.2 | 95.2 | 92.8 | 48.7 |

Abbreviations: AAR, AST to ALT ratio; APRI, AST to platelet ratio index; CI, confidence interval; EGD, esophagogastroduodenoscopy; Fib-4, Fibrosis-4; HVPG, hepatic venous pressure gradient; LSM, liver stiffness measurement; LSPS, LSM × spleen diameter to platelet ratio score; PSR, platelet count to spleen diameter ratio; SSM@50Hz, spleen stiffness measurement with liver mode; SSM@100Hz_,_ spleen stiffness measurement with spleen mode; VCTE, vibration controlled transient elastography

* Presence of GEV vs. non-GEV.

**SupplementaryTable 5.** Direct comparisons of the diagnostic accuracy of SSM@100Hz, SSM@50Hz, and other non-invasive tests in detecting GEV using EGD as the reference in the subgroup of patients with 10 valid measurements for all VCTE procedures

|  | N | AUROC | 95% CI | P value* | vs SSM@50Hz  P value | vs SSM@100Hz  P value | Cut-off level | Sensitivity | Specificity | PPV | NPV |
| --- | --- | --- | --- | --- | --- | --- | --- | --- | --- | --- | --- |
| **Elastography** |  | | | | | | | | | | |
| SSM@100Hz | 97 | 0.918 | 0.864-0.971 | < 0.001 | 0.237 | - | 37.1 | 86.0 | 87.2 | 87.7 | 85.4 |
| SSM@50Hz | 97 | 0.885 | 0.818-0.952 | < 0.001 | - | 0.237 | 24.4 | 94.0 | 70.2 | 77.0 | 91.6 |
| **Scoring systems** |  | | | | | | | | | | |
| AAR | 97 | 0.701 | 0.595-0.807 | < 0.001 | 0.008 | <0.001 | 1.29 | 78.0 | 61.7 | 68.4 | 72.5 |
| APRI | 97 | 0.686 | 0.579-0.793 | 0.001 | <0.001 | <0.001 | 0.844 | 62.0 | 74.4 | 72.0 | 64.8 |
| Fib-4 index | 97 | 0.716 | 0.615-0.818 | < 0.001 | 0.003 | <0.001 | 5.25 | 60.0 | 78.7 | 75.0 | 64.9 |
| PSR | 97 | 0.794 | 0.704-0.884 | < 0.001 | 0.043 | 0.003 | 969.4 | 68.0 | 82.9 | 80.9 | 70.9 |
| **Single marker** |  | | | | | | | | | | |
| Platelet count | 97 | 0.735 | 0.634-0.836 | < 0.001 | 0.005 | <0.001 | 95 | 56.0 | 85.1 | 80.0 | 64.5 |

Abbreviations: AAR, AST to ALT ratio; APRI, AST to platelet ratio index; CI, confidence interval; EGD, esophagogastroduodenoscopy; Fib-4, Fibrosis-4; GEV, gastroesophageal varices; LSM, liver stiffness measurement; LSPS, LSM-spleen diameter to platelet ratio score; PSR, platelet count to spleen diameter ratio; SSM@50Hz, spleen stiffness measurement with liver mode; SSM@100Hz, spleen stiffness measurement with spleen mode; VCTE, vibration controlled transient elastography

* Presence of GEV vs. non-GEV.

**SupplementaryTable 6.** Diagnostic accuracy of SSM@50Hz in detecting HRV using EGD as the reference in the subgroup of patients with 10 valid measurements for VCTE procedure

|  | N | AUROC | | Cut-off level | Sensitivity | Specificity | PPV | NPV |
| --- | --- | --- | --- | --- | --- | --- | --- | --- |
| **CAP ≤ 118 dB/m** |  | |  |  |  |  |  |  |
| SSM@50Hz | 77 | 0.801 | | 28.2 | 90.0 | 63.1 | 46.1 | 94.7 |
| **CAP > 118 dB/m** |  | |  |  |  |  |  |  |
| SSM@50Hz | 20 | 0.940 | | 70.2 | 80.0 | 90.0 | 88.8 | 81.8 |

Abbreviations: CAP, controlled attenuation parameter; EGD, esophagogastroduodenoscopy; SSM@50Hz, spleen stiffness measurement with liver mode; HRV, high-bleeding risk varices; VCTE, vibration controlled transient elastography

* Presence of GEV vs. non-GEV.

**Supplementary Table 7. Clinical and demographic characteristics of patients with CLD**

|  |  | Non-CSPH | |  | CSPH | |  |  |
| --- | --- | --- | --- | --- | --- | --- | --- | --- |
|  |  | N | Mean ± SD |  | N | Mean ± SD |  | P value |
| Age (years) |  | 9 | 50.1 ± 13.6 |  | 9 | 57.7 ± 8.4 |  | 0.171 |
| Sex (female: male) |  | 9 | 6 : 3 |  | 9 | 3 : 6 |  | 0.346 |
| Body mass index (kg/m^2^) |  | 9 | 24.3 ± 4.6 |  | 9 | 26.4 ± 4.6 |  | 0.349 |
| SCD (mm) |  | 9 | 18.7 ± 6.3 |  | 9 | 19.0 ± 4.7 |  | 0.904 |
| Alcohol/NAFLD/ Other |  | 9 | 1/1/7 |  | 9 | 1/6/2 |  | 0.025 |
| HCC (Yes/No) |  | 9 | 0 / 9 |  | 9 | 0 / 9 |  | 1.00 |
| Platelet count (10^9^/L) |  | 9 | 160 ± 80 |  | 9 | 107 ± 56 |  | 0.125 |
| PT (INR) |  | 9 | 1.11 ± 0.12 |  | 9 | 1.24 ± 0.23 |  | 0.140 |
| Albumin (g/dL) |  | 9 | 3.6 ± 0.7 |  | 9 | 3.2 ± 0.8 |  | 0.224 |
| Total bilirubin (mg/dL) |  | 9 | 1.2 ± 1.3 |  | 9 | 1.5 ± 0.8 |  | 0.584 |
| AST (U/L) |  | 9 | 24.1 ± 6.7 |  | 9 | 71.1 ± 61.2 |  | 0.036 |
| ALT (U/L) |  | 9 | 17.3 ± 8.9 |  | 9 | 56.2 ± 60.5 |  | 0.074 |
| Child-Pugh classification (A/B/C) |  | 9 | 5/3/1 |  | 9 | 4/2/3 |  | 0.689 |
| Spleen diameter (cm) |  | 9 | 11.2 ± 2.5 |  | 9 | 12.0 ± 2.4 |  | 0.473 |
| LSM (kPa) |  | 9 | 20.0 ± 16.6 |  | 8 | 36.6 ± 16.0 |  | 0.054 |
| SSM@50Hz (kPa) |  | 6 | 28.4 ± 5.6 |  | 6 | 57.4 ± 5.6 |  | 0.004 |
| SSM@100Hz (kPa) |  | 9 | 36.2 ± 16.3 |  | 9 | 61.5 ± 11.9 |  | 0.001 |
| HVPG (mmHg) |  | 9 | 5.5 ± 2.2 |  | 9 | 13.7 ± 4.1 |  | <0.001 |
| GEV (non/EV/GV/EV+GV/non) |  | 9 | 5/4/0/0 |  | 9 | 0/6/2/1 |  | 0.019 |

AST, aspartate aminotransferase; ALT, alanine aminotransferase; INR, international normalized ratio; LSM, liver stiffness measurement; PT, prothrombin time; HCC, hepatocellular carcinoma; SCD, skin capsule distance; SD, standard deviation; SSM50Hz, spleen stiffness measurement with liver mode; SSM@100Hz_,_ spleen stiffness measurement with spleen mode; CLD, chronic liver disease

**Supplementary Figure 1. Direct comparison of ROC curves of SSM@100Hz, SSM@50Hz, and LSM for identifying GEV in patients with CLD**


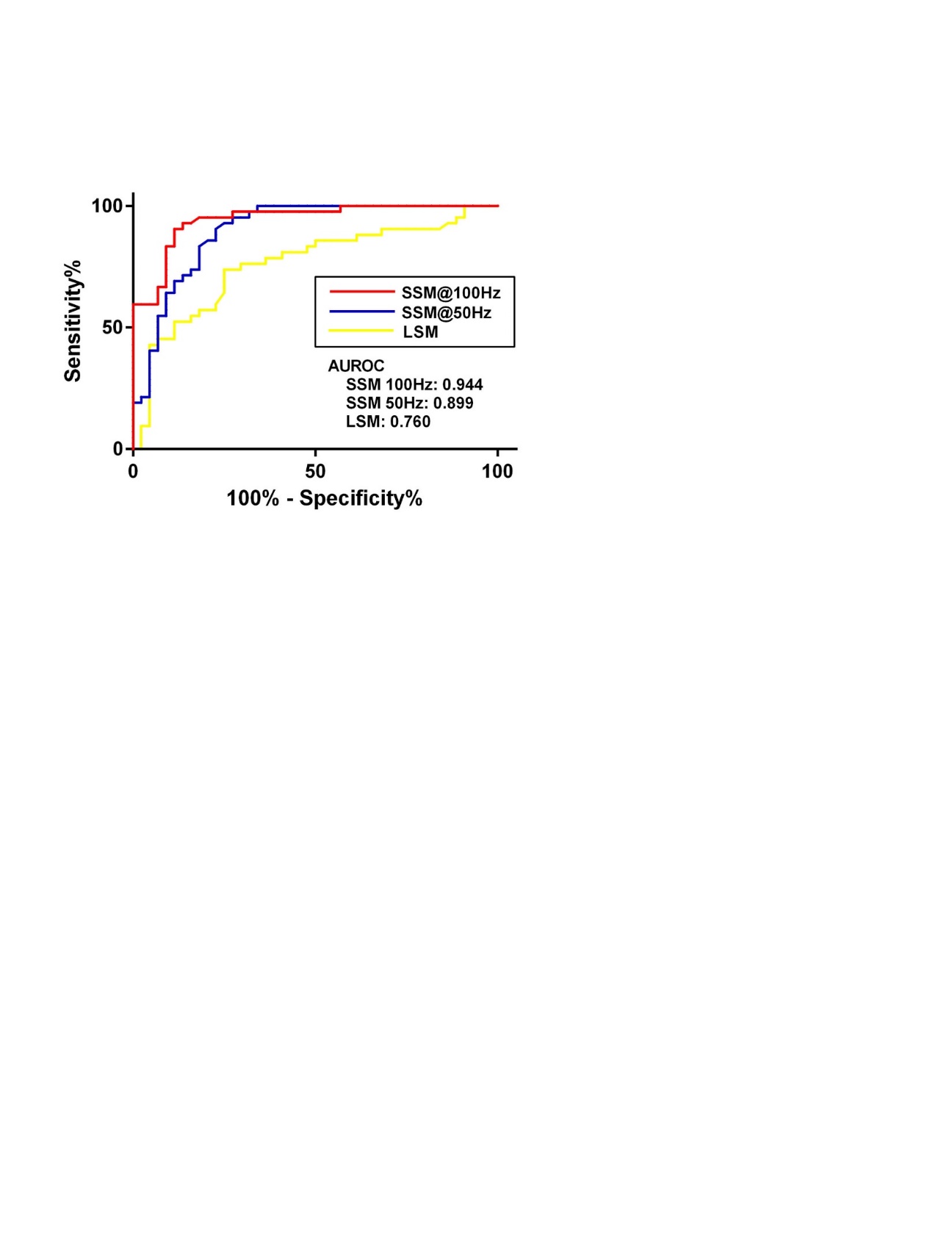


CLD, chronic liver disease; GEV, gastroesophageal varices; LSM, liver stiffness measurement; SSM@50Hz, spleen stiffness measurement with liver mode; SSM@100Hz, spleen stiffness measurement with spleen mode; ROC, receiver operating characteristic.

**Supplementary Figure 2. Direct comparison of ROC curves of SSM@100Hz, SSM@50Hz, and LSM for identifying GEV in patients with liver cirrhosis**


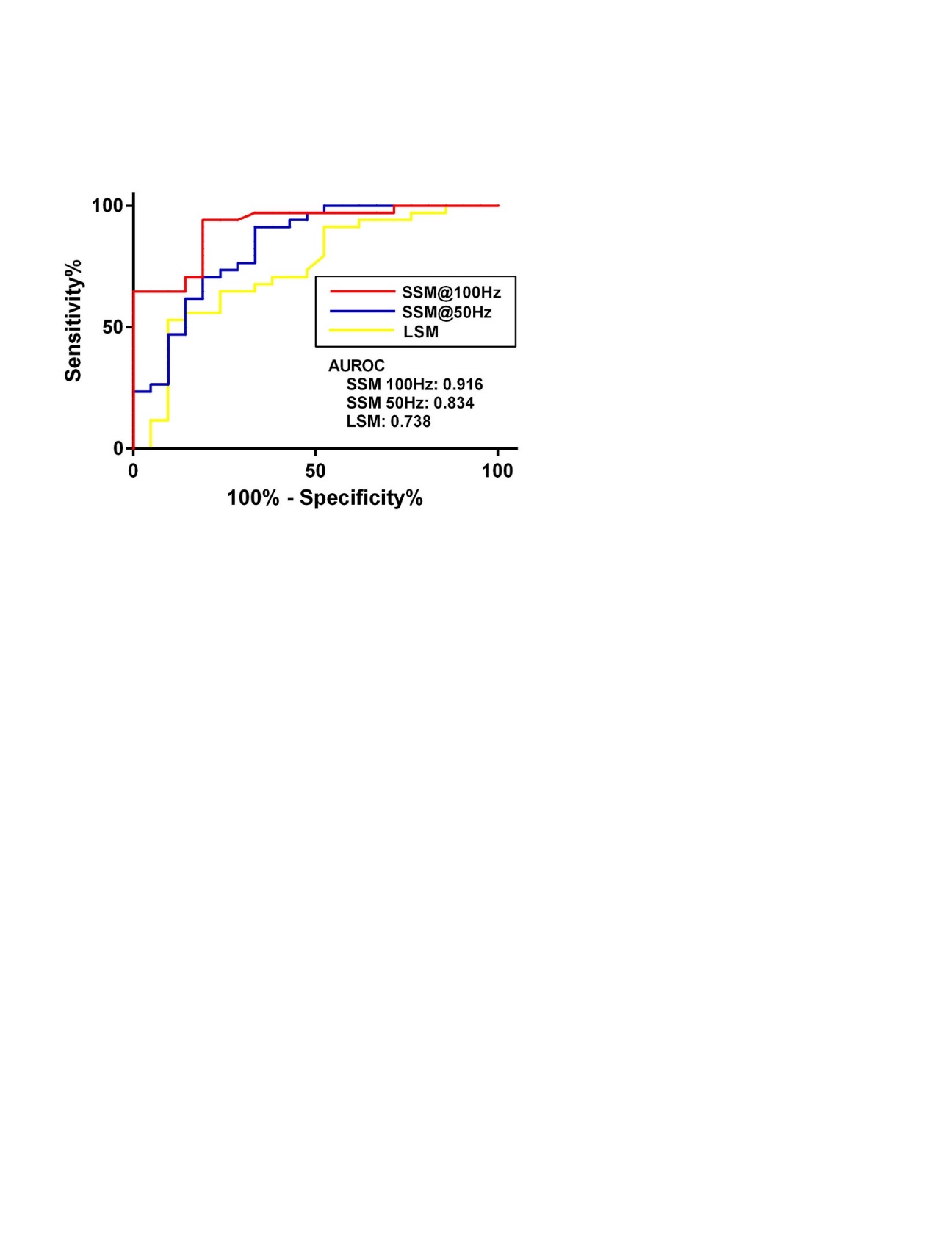


GEV, gastroesophageal varices; LSM, liver stiffness measurement; SSM@50Hz, spleen stiffness measurement with liver mode; SSM@100Hz, spleen stiffness measurement with spleen mode; ROC, receiver operating characteristic.

**Supplementary Figure 3. Distribution and direct comparison of ROC curves between SSM@100Hz and SSM@50Hz for identifying GEV in patients with CLD**


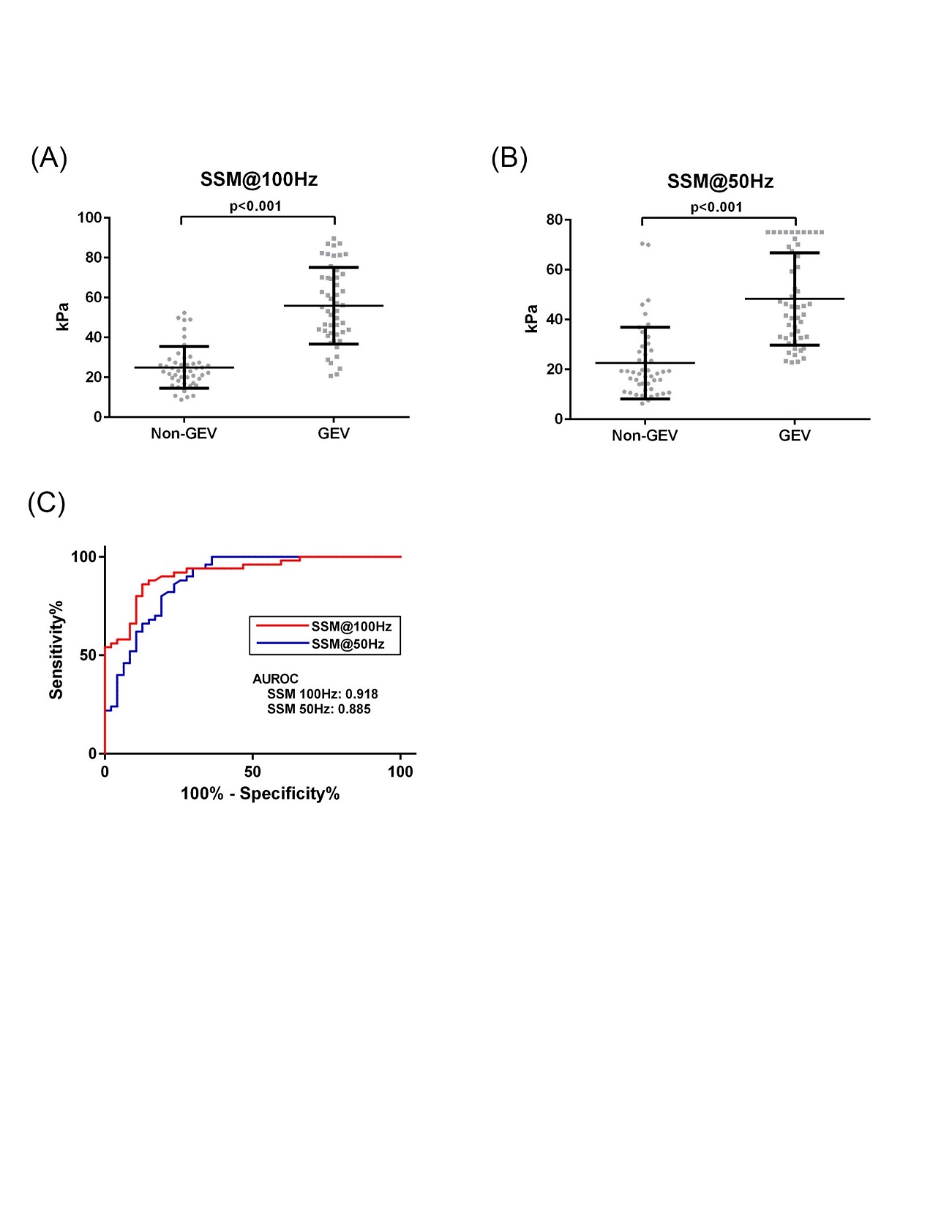


Distribution of (A) SSM@100Hz and (B) SSM@50Hz among CLD patients with and without GEV. (C) Direct comparison of ROC curves of SSM@100Hz and SSM@50Hz for identifying GEV.

CLD, chronic liver disease; GEV, gastroesophageal varices; SSM@50Hz, spleen stiffness measurement with liver mode; SSM@100Hz, spleen stiffness measurement with spleen mode; ROC, receiver operating characteristic.

**Supplementary Figure 4. Correlation between SSM@100Hz and SSM@50Hz**

**
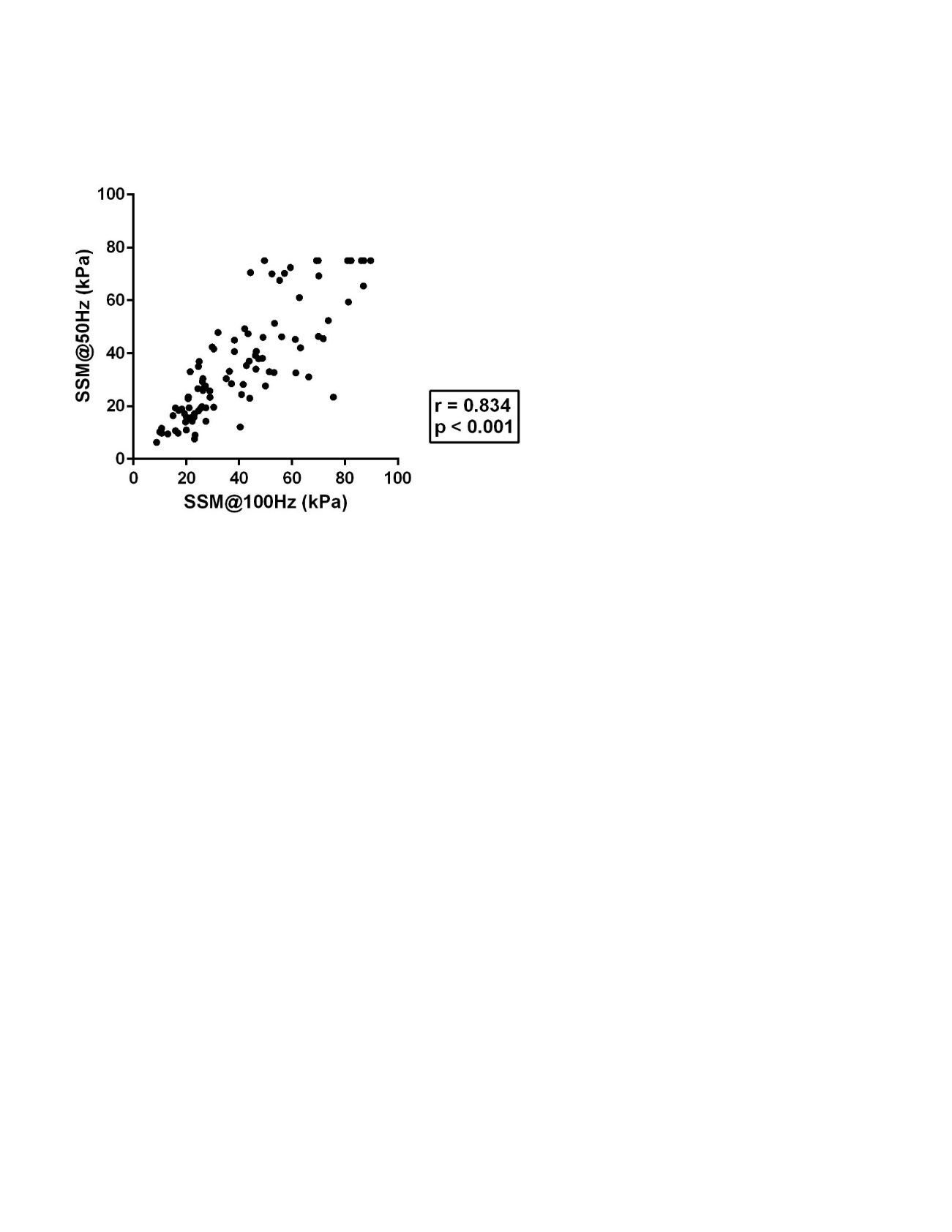
**

SSM@50Hz, spleen stiffness measurement with liver mode; SSM@100Hz, spleen stiffness measurement with spleen mode

**Supplementary Figure 5. Distribution between SSM@100Hz and SSM@50Hz with EV grade and GV grade in the patients with CLD**


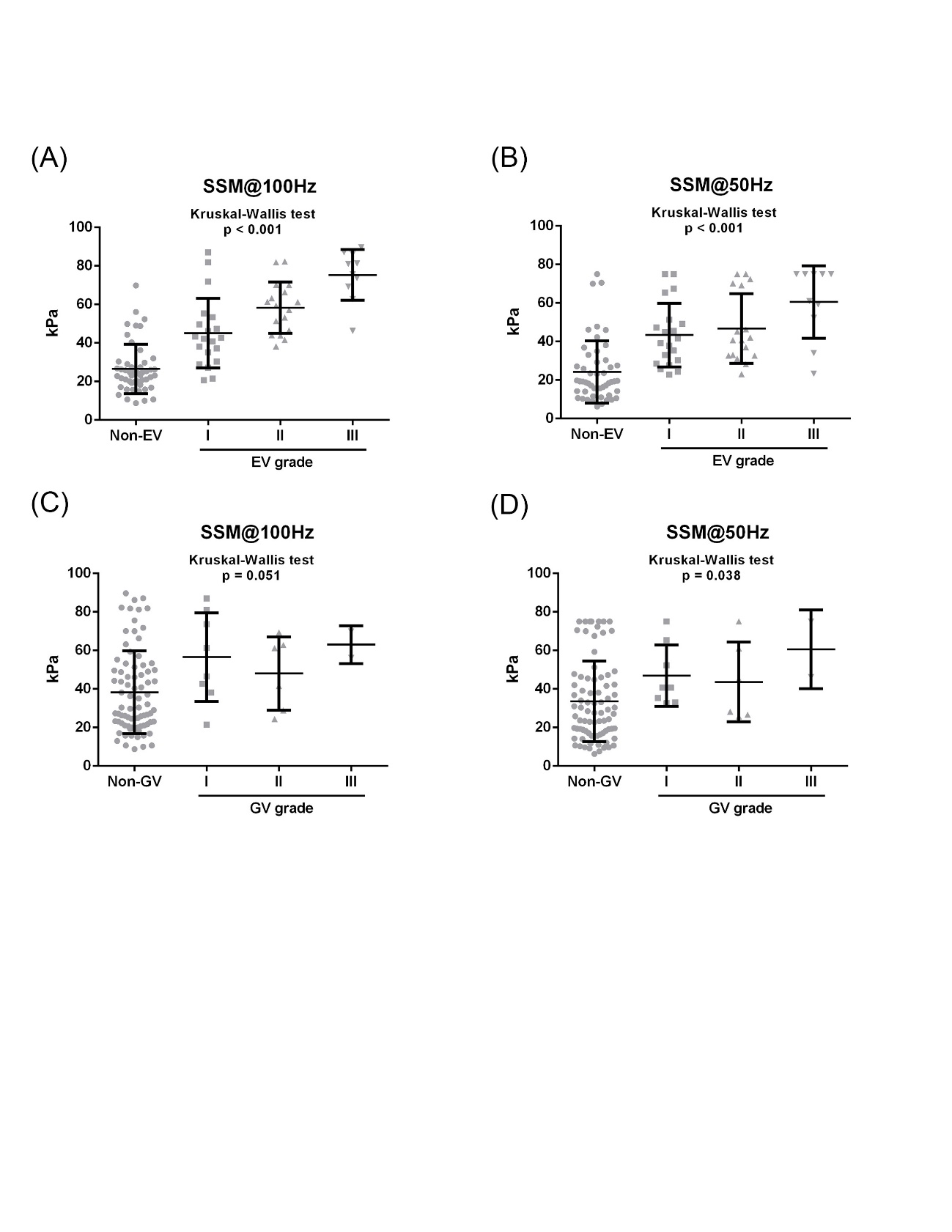


(A) Distribution of SSM@100Hz with EV grade in patients with CLD. The median values for SSM@100Hz was 26.5 ± 12.7 kPa, non-EV (n=50); 45.0 ± 18.0 kPa, grade I (n=20); 58.3 ± 13.3kPa, grade II (n=17); 75.3 ± 13.2 kPa, grade III (n=10) (Kruskal-Wallis test, P<0.001).

(B) Distribution of SSM@50Hz with EV grade in patients with CLD. The median values for SSM@50Hz was 24.1 ± 16.1 kPa, non-EV (n=50); 43.3 ± 16.4 kPa, grade I (n=20); 46.7 ± 18.1 kPa, grade II (n=17); 60.5 ± 18.8 kPa, grade III (n=10) (Kruskal-Wallis test, P<0.001).

(C) Distribution of SSM@100Hz with GV grade in patients with CLD. The median values for SSM@100Hz was 38.3 ± 21.4 kPa, non-GV (n=81); 56.5 ± 22.9 kPa, grade I (n=8); 48.0 ± 19.0 kPa, grade II (n=6); 63.0 ± 9.7 kPa, grade III (n=2) (Kruskal-Wallis test, P=0.051).

(D) Distribution of SSM@50Hz with GV grade in patients with CLD. The median values for SSM@50Hz was 33.5 ± 20.9 kPa, non-GV (n=81); 46.8 ± 15.9 kPa, grade I (n=8); 43.6 ± 20.6 kPa, grade II (n=6); 60.6 ± 20.3 kPa, grade III (n=2) (Kruskal-Wallis test, P=0.038).

CLD, chronic liver disease; EV, esophageal varices; GV, gastric varices; SSM@50Hz, spleen stiffness measurement with liver mode; SSM@100Hz, spleen stiffness measurement with spleen mode

**Supplementary Figure 6. Distribution of SSM@100Hz and SSM@50Hz between RC- GEV group and RC+ GEV group in patients with CLD**


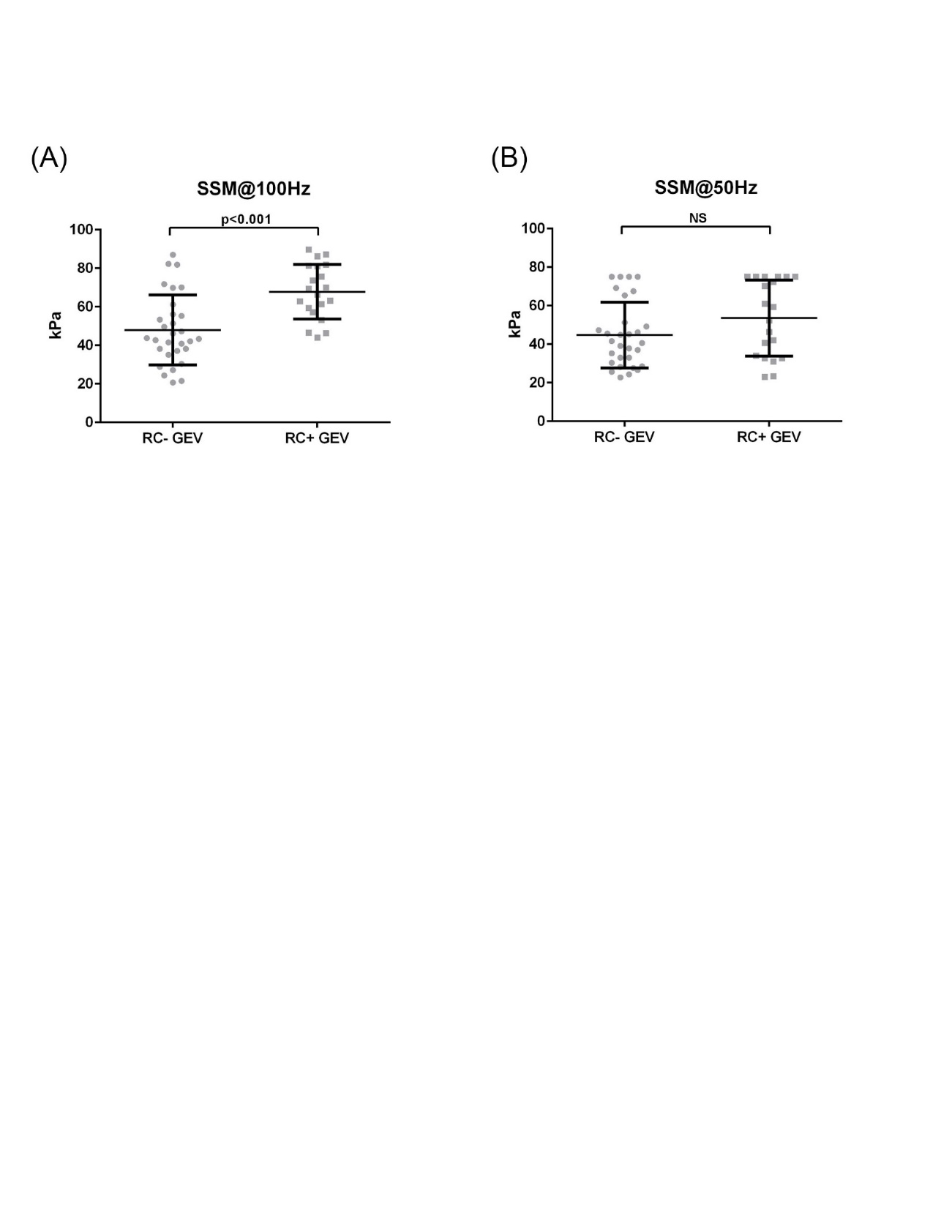


(A) Distribution of SSM@100Hz between RC- GEV group and RC+ GEV group in CLD patients with EV and/or GV of grade ≥ I (n=50). The median values for SSM@100Hz of among those GEV who were positive for red color sign (RC+) (67.7 ± 14.1 kPa, n=20) were significantly higher than those with GEV who negative for red color sign (RC-) (47.9 ± 18.1 kPa, n=30) (p<0.001).

(B) Distribution of SSM@50Hz between RC- GEV group and RC+ GEV group in CLD patients with EV and/or GV of grade ≥ I (n=50). The median values for SSM@50Hz did not show significant difference between those with GEV who were RC+ (53.3 ± 19.7kPa, n=20) and those with GEV who were RC- (44.7 ± 17.0 kPa, n=30) (p=0.101).

CLD, chronic liver disease; GEV, gastroesophageal varices; RC, red color sign; SSM@50Hz, spleen stiffness measurement with liver mode; SSM@100Hz, spleen stiffness measurement with spleen mode
